# Weakly coupled bound state of 2-D Schrödinger operator with potential-measure

**DOI:** 10.1016/j.jmaa.2014.06.053

**Published:** 2014-12-15

**Authors:** Sylwia Kondej, Vladimir Lotoreichik

**Keywords:** Schrödinger operator, Perturbations by measures, Eigenvalues, Bound states

## Abstract

We consider a self-adjoint two-dimensional Schrödinger operator $H_{\alpha \mu }$, which corresponds to the formal differential expression

−Δ−αμ,

where *μ* is a finite compactly supported positive Radon measure on $R^{2}$ from the generalized Kato class and α>0 is the coupling constant. It was proven earlier that $\sigma_{\text{ess}}(H_{\alpha \mu })=[0,+\infty )$. We show that for sufficiently small *α* the condition $♯\sigma_{d}(H_{\alpha \mu })=1$ holds and that the corresponding unique eigenvalue has the asymptotic expansion

$$\lambda (\alpha )=-\left(C_{\mu }+o(1)\right)\exp\left(-\frac{4\pi }{\alpha \mu (R^{2})}\right),\;\alpha \to 0+,$$

with a certain constant $C_{\mu }>0$. We also obtain a formula for the computation of $C_{\mu }$. The asymptotic expansion of the corresponding eigenfunction is provided. The statements of this paper extend the results of Simon [41] to the case of potentials-measures. Also for regular potentials our results are partially new.

## 1 Introduction

Let us consider a non-relativistic quantum particle living in a two-dimensional system and moving under the influence of the potential $V:R^{2}\to R$ such that there exists δ>0 for which

(1.1) $$\int_{R^{2}}{\left|V(x)\right|}^{1+\delta }<\infty \;\text{and}\;\int_{R^{2}}\left|V(x)\right|\left(1+{\left|x\right|}^{\delta }\right)<\infty .$$

The closed, densely defined, symmetric and lower-semibounded sesquilinear form

$$t_{\alpha V}[f,g]:={\left(\nabla f,\nabla g\right)}_{L^{2}(R^{2};C^{2})}-\alpha {\left(Vf,g\right)}_{L^{2}(R^{2})},\;\mathrm{dom}\;t_{\alpha V}:=H^{1}\left(R^{2}\right),$$

induces the self-adjoint Hamiltonian $H_{\alpha V}$ in $L^{2}(R^{2})$. The spectrum of $H_{\alpha V}$ cannot be computed explicitly for an arbitrary potential. For this reason spectral estimates and asymptotic expansions of spectral quantities related to $H_{\alpha V}$ attract a lot of attention. Weak coupling asymptotic regime belongs to this line of research. It was shown by Simon in [41] that under the assumptions

(1.2) $$\int_{R^{2}}V(x)\geq 0\;\text{and}\;V≢0$$

the operator $H_{\alpha V}$ has at least one bound state for any α>0; moreover in the limit α→0+ the corresponding lowest eigenvalue asymptotically behaves as

(1.3) $$\lambda (\alpha )∼\exp\left({\left[-\frac{\alpha }{4\pi }\int_{R^{2}}V(x)\right]}^{-1}\right),\;\alpha \to 0+,$$

provided that inequality (1.2) is strict; cf. [41, Theorem 3.4]. The problem we study in this paper, is addressed in a certain respect to a more general class of potentials which, for example, includes so-called *singular interactions*. To sketch the physical context suppose that a particle is confined by a quantum wire with possibility of tunnelling. Consequently, the whole space $R^{2}$ is available for the particle. On the other hand, if the wire is very thin, we can make an idealization and assume that the particle is localized in the vicinity of the set $\Sigma \subset R^{2}$ of a co-dimension one. The Hamiltonian of such a system can be formally written as

$$-\Delta -\alpha \delta_{\Sigma },\;\alpha >0,$$

where $\delta_{\Sigma }$ denotes the Dirac measure supported on *Σ*, see [18] for the review on such Hamiltonians. More generally, one can speak of

−Δ−αμ,α>0,

where *μ* is a positive finite Radon measure on $R^{2}$. In order to give a mathematical meaning to the above formal expression we assume that *μ* belongs to the generalized Kato class as in Definition 2.1. Under this assumption the embedding of $H^{1}(R^{2})$ into $L^{2}(R^{2};d\mu )$ is well defined and the following closed, densely defined, symmetric and lower-semibounded sesquilinear form

$$t_{\alpha \mu }[f,g]:={\left(\nabla f,\nabla g\right)}_{L^{2}(R^{2};C^{2})}-\alpha \int_{R^{2}}f(x)\bar{g(x)}d\mu (x),\;\mathrm{dom}\;t_{\alpha \mu }:=H^{1}\left(R^{2}\right),$$

induces the uniquely defined self-adjoint operator $H_{\alpha \mu }$ in $L^{2}(R^{2})$. It is known that $\sigma_{\mathrm{ess}}(H_{\alpha \mu })=[0,+\infty )$, see [12, Theorem 3.1]. The following theorem contains all the main results of the paper.

Theorem *Let μ be a compactly supported positive finite Radon measure on* $R^{2}$ *from the generalized Kato class and* $H_{\alpha \mu }$ *be the self-adjoint operator defined above. Then the following statements hold.*

(i) *For* α>0 *sufficiently small* $♯\sigma_{d}(H_{\alpha \mu })=1$ *. Denote this unique eigenvalue by* λ(α)<0 *and the corresponding eigenfunction by* $f_{\alpha }\in L^{2}(R^{2})$ *.*
(ii) *The asymptotic expansion of* λ(α) *takes the form*
  $$\lambda (\alpha )=-\left(C_{\mu }+o(1)\right)\exp\left(-\frac{4\pi }{\alpha \mu (R^{2})}\right),\;\alpha \to 0+,$$
  *where the constant* $C_{\mu }$ *is given in* (3.3) *.*
(iii) *Set* $k_{\alpha }={\left(-\lambda (\alpha )\right)}^{1/2}$ *. Then the corresponding eigenfunction admits the following expansion*
  $$f_{\alpha }(\cdot )=\frac{k_{\alpha }}{2\pi }\int_{R^{2}}K_{0}\left(k_{\alpha }|\cdot -y|\right)d\mu (y)+O\left(\frac{1}{\ln k_{\alpha }}\right),\;\alpha \to 0+,$$
  *where* $K_{0}(\cdot )$ *is the Macdonald function, the norm of the first summand has non-zero finite limit as* α→0+ *, and the error term is understood in the strong sense.*

The reader may note that in the asymptotic expansion of λ(α) the dominating term depends only on the total measure of $R^{2}$ and does not depend on the distribution character of the measure *μ*. This stays in consistency with the result of Simon and reflects the property that in the weak coupling regime spectral quantities “forget” about local properties of the potential.

The statements of this paper complement and extend the results of [41] for the generalized class of potentials given by Radon measures. Firstly, the class of perturbations that we admit contains, for example, singular measures as *δ*-distributions supported on sets of co-dimension one. However, let us note that a class of perturbations supported by sets with a fractional Hausdorff dimension is admissible as well, cf. [3]. Secondly, for regular compactly supported potentials our class is slightly larger than that of [41]. In order to give the reader an idea of that, let us only mention that radially symmetric potential

$$V(r)=\frac{\chi (r)}{r^{2}{\left|\ln(r)\right|}^{\gamma }},$$

with χ(r) being the characteristic function of the interval [0,1/2] and γ>2, is compactly supported and belongs to the generalized Kato class, however it does not satisfy assumptions (1.1), which are imposed in [41]. One should say that the formula for the constant $C_{\mu }$ given in (3.3) was derived formally by physicists [37] for the case of regular potentials, but without a rigorous mathematical proof. It appears also in the paper [8], but again for a regular class of potentials.

Analogous asymptotic expansions of the bound state with respect to a small parameter appear in various spectral problems. It is worth to mention such results for two-dimensional waveguides with weak local perturbations [13] as well as for coupled waveguides with a small window [38] and also with a semi-transparent window [23]. Recently a “leaky waveguide” with a small parameter breaking the symmetry was considered in [31]. For the similar problems in the one-dimensional case see [10], [29], [36], [41]. The analogous results for quantum graphs were obtained in [16], [17], [35]. See also recent developments for Pauli operators [25]. Our list of references is by no means complete, however many of closely related works are mentioned.

In order to prove the main statements we apply the Birman–Schwinger principle. Precisely saying, we use its generalization for potentials-measures from the generalized Kato class, which is rigorously established in [12], see also [11] and [5], [39] for further modifications. We also use some simple results of perturbation theory of linear operators, where the standard reference is [28], however we require some extensions of the classical results.

Extensions of our results to non-compactly supported finite Kato class measures are also discussed. Using purely variational arguments combined with our main result for compactly supported measures we are able to obtain weak coupling asymptotics of the lowest eigenvalue for a class of non-compactly supported measures, which is in some respects wider also for regular potentials than the one considered in [8], [41]. In particular, in Example 4.1 we construct a regular potential, for which our methods are applicable, but which does not satisfy the second condition in (1.1).

The paper is organized as follows. In Section 2 we complete some mathematical tools useful for further spectral analysis. Namely, we provide a rigorous definition of the Hamiltonian $H_{\alpha \mu }$, formulate the Birman–Schwinger principle, develop a perturbation method for a particular class of non-analytic operator families and analyze the properties of the operators involved into the Birman–Schwinger principle. In Section 3 we formulate and prove main results of the paper concerning the uniqueness of the bound state in the weak coupling regime, obtain its asymptotics and derive the behavior of the corresponding eigenfunction. Section 4 is devoted to a discussion on non-compactly supported measures.

The following abbreviations are used throughout the body of the paper:

• we set $L^{2}:=L^{2}(R^{2})$, $L^{1}:=L^{1}(R^{2})$, $H^{k}:=H^{k}(R^{2})$ with k∈Z (norm ${\left\|\cdot \right\|}_{k}$) and $L^{2}:=L^{2}(R^{2};C^{2})$;
• the notation $S:=S(R^{2})$ stands for the Schwartz class, moreover we set $S^{\prime }:=S^{\prime }(R^{2})$ for the space dual to S, i.e. $S^{\prime }$ is the space of linear continuous functionals on S;
• we set $L_{\mu }^{2}:=L^{2}(R^{2};d\mu )$, $L^{1}(R^{2};d\mu )$, and $L_{\mu }^{\infty }:=L^{\infty }(R^{2};d\mu )$;
• for the positive Radon measure *μ* on $R^{2}$ we denote $\mu_{T}:=\mu (R^{2})$.

## 2 Preliminaries

This section plays an auxiliary role and consists of four subsections. In Section 2.1 we provide necessary facts from [11], [12] on self-adjoint free Laplacians perturbed by Kato-class measures. In Section 2.2 we prove some statements on non-analytic perturbation theory, which are hard to find in the literature. In Sections 2.3, 2.4 we complement known results on the operators related to the Birman–Schwinger principle.

### 2.1 Self-adjoint Laplacians perturbed by Kato-class measures

We start by recalling the definition of the generalized Kato class of positive Radon measures on $R^{2}$.

Definition 2.1 A positive Radon measure *μ* on $R^{2}$ belongs to the generalized Kato class if

$$\lim_{\varepsilon \to 0+}\underset{x\in R^{2}}{\sup}\int_{D_{\varepsilon }(x)}\left|\ln\left(|x-y|\right)\right|d\mu (y)=0,$$

where $D_{\varepsilon }(x)$ is the disc of radius ε>0 with the center at $x\in R^{2}$.

Let *μ* be a positive Radon measure from the generalized Kato class. Then for arbitrarily small ε>0 there exists a constant C(ε)>0 such that

$$\int_{R^{2}}{\left|f(x)\right|}^{2}d\mu (x)\leq \varepsilon {\left\|\nabla f\right\|}_{L^{2}}^{2}+C(\varepsilon ){\left\|f\right\|}_{L^{2}}^{2}$$

holds for every f∈S; see [12], [42]. For the measure *μ* the embedding operator $J_{\mu }:H^{1}\to L_{\mu }^{2}$ is well-defined as the closure of the natural embedding defined on the Schwartz class, see [12, Section 2]. Consequently, the above inequality has a natural extension, i.e. for arbitrarily small ε>0 there exists a constant C(ε)>0 such that

(2.1) $${\left\|J_{\mu }f\right\|}_{L_{\mu }^{2}}^{2}\leq \varepsilon {\left\|\nabla f\right\|}_{L^{2}}^{2}+C(\varepsilon ){\left\|f\right\|}_{L^{2}}^{2},$$

for all $f\in H^{1}$.

Example 2.1 Suppose that the measurable function $V:R^{2}\to [0,+\infty )$ satisfies the condition

$$\lim_{\varepsilon \to 0+}\underset{x\in R^{2}}{\sup}\int_{D_{\varepsilon }(x)}\left|\ln\left(|x-y|\right)\right|V(y)dy=0.$$

Then the measure

$$\mu_{V}(\Omega ):=\int_{\Omega }V(x)dx$$

belongs to the generalized Kato class.

Example 2.2 (See [44, Example 2.3(c)].) Given a family ${\left\{\Gamma_{i}\right\}}_{i=1}^{N}$ of Lipschitz curves in the plane. Suppose that each curve in the family is parameterized by its arc length $\gamma_{i}:[0,|\Gamma_{i}|]\to R^{2}$ and $\gamma_{i}([0,|\Gamma_{i}|])=\Gamma_{i}$ with i=1,2,…,N. Assume that for all $s,t\in [0,|\Gamma_{i}|]$ and every i=1,2,…,N the condition $\left|\gamma_{i}(s)-\gamma_{i}(t)|\geq 1/2|s-t\right|$ holds. So that each curve cannot have cusps and cannot intersect itself, whereas different curves can intersect each other. Now let $\Gamma :={⋃}_{i=1}^{N}\Gamma_{i}$. Then the Dirac measure supported on *Γ* belongs to the generalized Kato class.

Let the self-adjoint operator

$$-\Delta_{\text{free}}f:=-\Delta f,\;\mathrm{dom}(-\Delta_{\text{free}}):=H^{2},$$

define the unperturbed Hamiltonian of our system. In fact, $-\Delta_{\text{free}}$ represents closed, densely defined, symmetric and lower-semibounded sesquilinear form

(2.2) $$t[f,g]={\left(\nabla f,\nabla g\right)}_{L^{2}},\;\mathrm{dom}\;t=H^{1}.$$

Let *μ* be a positive Radon measure from the generalized Kato class. By means of *μ* we define the sesquilinear form

(2.3) $$t_{\alpha \mu }[f,g]:=t[f,g]-{\left(\alpha J_{\mu }f,J_{\mu }g\right)}_{L_{\mu }^{2}},\;\mathrm{dom}\;t_{\alpha \mu }:=H^{1},$$

which in view of (2.1) and KLMN-theorem, cf. [40, Theorem X.17], is closed, densely defined, symmetric and lower-semibounded in $L^{2}$.

Definition 2.2 Let $H_{\alpha \mu }$ be a self-adjoint operator acting in $L^{2}$ and defined as the operator associated with $t_{\alpha \mu }$ via the first representation theorem, [28, Chapter VI, Theorem 2.1].

Denote $R(\lambda ):={\left(-\Delta_{\text{free}}-\lambda \right)}^{-1}$ with $\lambda \in C\setminus R_{+}$. Then R(λ) is an integral operator with the kernel

$$G(x,y;\lambda )=\frac{1}{2\pi }K_{0}(\sqrt{-\lambda }|x-y|),\;x,y\in R^{2},$$

where $K_{0}(\cdot )$ is the Macdonald function, see [1, §9.6]. Following the notations of [12] we introduce the integral operator

(2.4) $$R_{\mu dx}(\lambda ):L_{\mu }^{2}\to L^{2},\;R_{\mu dx}f:=\int_{R^{2}}G(x,y;\lambda )f(y)d\mu (y),$$

and define the “bilateral” embedding of R(λ) to $L_{\mu }^{2}$ by

(2.5) $$Q(\lambda ):=J_{\mu }R_{\mu dx}(\lambda ):L_{\mu }^{2}\to L_{\mu }^{2}.$$

Note that

(2.6) $$Q\left(-k^{2}\right)f:=\frac{1}{2\pi }\int_{R^{2}}K_{0}\left(k|\cdot -y|\right)f(y)d\mu (y).$$

The Birman–Schwinger principle has the following form.

Proposition 2.3 *(See* *[11, Lemma 1]* *,* *[12]* *.) Let* $R_{\mu dx}(\cdot )$ *,* Q(⋅) *and* $H_{\alpha \mu }$ *be as above. For* $\lambda \in R_{-}$ *the mapping*

$$h\mapsto R_{\mu dx}(\lambda )h$$

*is a bijection from* ker⁡(I−αQ(λ)) *onto* $\ker(H_{\alpha \mu }-\lambda )$ *, and*

$$\dim\ker\left(I-\alpha Q(\lambda )\right)=\dim\ker(H_{\alpha \mu }-\lambda ).$$

We will also use the fact that the essential spectrum is stable under a perturbation by a finite measure.

Proposition 2.4 *(See* *[12, Theorem 3.1]* *.) Let μ be a positive Radon measure on* $R^{2}$ *from the generalized Kato class. Assume that* $\mu_{T}<\infty$ *and* $H_{\alpha \mu }$ *is as in* Definition 2.2 *. Then*

$$\sigma_{\text{ess}}(H_{\alpha \mu })=[0,+\infty )$$

*holds.*

Remark 2.5 Note that also more singular perturbations are considered. For example, *δ*-interactions supported on curves in $R^{3}$, see e.g. [20], [21], [22], [30], [39], and $\delta^{\prime }$-interactions supported on hypersurfaces, see e.g. [5], [6], [19]. Point *δ*-interactions in $R^{d}$ with d=2,3 are also more singular, cf. [4]. These perturbations do not belong to the generalized Kato class and therefore they require different approaches.

### 2.2 Elements of non-analytic perturbation theory

For later purposes we analyze a family of self-adjoint operators k↦T(k), $k\in R_{+}$, acting in a Hilbert space H and taking the form

$$T(k):=T_{0}+\frac{1}{\ln k}T_{1}+O\left(\frac{1}{\ln^{2}k}\right),\;k\to 0+,$$

where $T_{0}=\varphi (\cdot ,\varphi )$ with φ∈H such that ‖φ‖=1, $T_{1}$ is a bounded self-adjoint operator in H and the error term is understood in the operator norm sense. The family T(⋅) is not analytic and consequently we cannot apply directly the results of [28, Chapters II and VII]. In the following theorem we investigate the spectra and the eigenfunctions of T(k) in the limit k→0+.

Theorem 2.6 *Let* k↦T(k) *be defined as above. For sufficiently small* k>0 *the spectrum* σ(T(k))⊂R *of* T(k) *consists of two disjoint components* $\sigma_{0}(k)$ *and* $\sigma_{1}(k)$ *.*

(i) *The part* $\sigma_{0}(k)$ *is located in the small neighborhood of zero and its diameter can be estimated as*
  $$\mathrm{diam}\;\sigma_{0}(k)\leq \frac{1}{\left|\ln k\right|}\|T_{1}\|+O\left(\frac{1}{\ln^{2}k}\right),\;k\to 0+.$$
(ii) *The part* $\sigma_{1}(k)$ *consists of exactly one eigenvalue* ω(k) *of multiplicity one, which depends on k continuously.*
(iii) *The normalized eigenfunction* $\varphi_{k}$ *corresponding to the eigenvalue* ω(k) *has the following expansion*
  (2.7) $$\varphi_{k}=\varphi +O\left(\frac{1}{\ln k}\right),\;k\to 0+,$$
  *in the norm of* H *.*
(iv) *The eigenvalue* ω(k) *has the asymptotics*
  (2.8) $$\omega (k)=1+\frac{1}{\ln k}(T_{1}\varphi ,\varphi )+O\left(\frac{1}{\ln^{2}k}\right),\;k\to 0+.$$

Proof (i) Note that $\sigma (T_{0})=\{0,1\}$ and that *φ* is an eigenfunction of the operator $T_{0}$ corresponding to the eigenvalue 1. The separation of the spectra of T(k) into two parts $\sigma_{0}(k)$ and $\sigma_{1}(k)$ for sufficiently small k>0 follows from [28, Theorem V.4.10]. The component $\sigma_{0}(k)$ is located in the neighborhood of 0 and the component $\sigma_{1}(k)$ is located in the neighborhood of 1. Note that again by [28, Theorem V.4.10] the diameter of $\sigma_{0}(k)$ satisfies

$$\mathrm{diam}\;\sigma_{0}(k)\leq \frac{1}{\left|\ln k\right|}\|T_{1}\|+O\left(\frac{1}{\ln^{2}k}\right),\;k\to 0+.$$

(ii) Let $E_{i}(k)$, i=0,1, be the orthogonal projectors onto the spectral subspaces of the operator T(k) corresponding to $\sigma_{i}(k)$. Then $E_{0}(0)=I-T_{0}$ and $E_{1}(0)=T_{0}$ hold. Since $\left\|T(k)-T_{0}\right\|$ tends to 0 for k→0+, relying on [15, Theorem 3], we have $\dim\mathrm{ran}\;E_{1}(k)=1$ for sufficiently small k>0. Therefore $E_{1}(k)={\overset{˜}{\varphi }}_{k}(\cdot ,{\overset{˜}{\varphi }}_{k})$, where ${\overset{˜}{\varphi }}_{k}$ is the normalized eigenfunction corresponding to the eigenvalue ω(k) of T(k) with multiplicity one. According to [28, Theorem VIII.1.14] the eigenvalue ω(k) depends on *k* continuously.

(iii) By [34, Proposition 2.1], see also [9], the estimate

$$\mathrm{dist}\;\left(\sigma_{0}(k),\sigma_{1}(k)\right)\left\|E_{0}(k)E_{1}(0)\right\|\leq \frac{\pi }{2}\left\|T(k)-T(0)\right\|$$

holds, which yields the asymptotic property

$$\left\|E_{1}(0)-E_{1}(k)E_{1}(0)\right\|=O\left(\frac{1}{\ln k}\right),\;k\to 0+,$$

where we have used $E_{0}(k)=I-E_{1}(k)$. The above expansion implies the following

(2.9) $$\left\|\varphi -{\overset{˜}{\varphi }}_{k}(\varphi ,{\overset{˜}{\varphi }}_{k})\right\|=O\left(\frac{1}{\ln k}\right),\;k\to 0+.$$

A straightforward calculation yields

$${\left\|\varphi -{\overset{˜}{\varphi }}_{k}(\varphi ,{\overset{˜}{\varphi }}_{k})\right\|}^{2}=\left(\varphi -{\overset{˜}{\varphi }}_{k}(\varphi ,{\overset{˜}{\varphi }}_{k}),\varphi -{\overset{˜}{\varphi }}_{k}(\varphi ,{\overset{˜}{\varphi }}_{k})\right)=1-({\overset{˜}{\varphi }}_{k},\varphi )(\varphi ,{\overset{˜}{\varphi }}_{k})-(\varphi ,{\overset{˜}{\varphi }}_{k})\bar{\left(\varphi ,{\overset{˜}{\varphi }}_{k}\right)}+{\left|(\varphi ,{\overset{˜}{\varphi }}_{k})\right|}^{2}=1-{\left|({\overset{˜}{\varphi }}_{k},\varphi )\right|}^{2}.$$

Combining the above result with the estimate (2.9) we arrive at

(2.10) $$1-{\left|({\overset{˜}{\varphi }}_{k},\varphi )\right|}^{2}=O\left(\frac{1}{\ln^{2}k}\right),\;k\to 0+.$$

Consequently, we obtain

(2.11) $$1-\left|({\overset{˜}{\varphi }}_{k},\varphi )\right|=O\left(\frac{1}{\ln^{2}k}\right),\;k\to 0+.$$

Suppose that (r(k),θ(k)) determine the polar representation of $\left({\overset{˜}{\varphi }}_{k},\varphi \right)$, i.e. $({\overset{˜}{\varphi }}_{k},\varphi )=r(k)e^{i\theta (k)}$, where r(k)>0. According to (2.11) we claim that

(2.12) $$r(k)=1+O\left(\frac{1}{\ln^{2}k}\right),\;k\to 0+.$$

Since ${\overset{˜}{\varphi }}_{k}$ is the normalized eigenfunction of T(k) corresponding to the eigenvalue ω(k) the function

(2.13) $$\varphi_{k}:=e^{i\theta (k)}{\overset{˜}{\varphi }}_{k}$$

is as well. Thence, by (2.9) and (2.12) we get

$$\|\varphi -\varphi_{k}\|=\left\|\varphi -e^{i\theta (k)}{\overset{˜}{\varphi }}_{k}\right\|\leq \left\|\varphi -r(k)e^{i\theta (k)}{\overset{˜}{\varphi }}_{k}\right\|+\left\|r(k)e^{i\theta (k)}{\overset{˜}{\varphi }}_{k}-e^{i\theta (k)}{\overset{˜}{\varphi }}_{k}\right\|=\left\|\varphi -(\varphi ,{\overset{˜}{\varphi }}_{k}){\overset{˜}{\varphi }}_{k}\right\|+\left|r(k)-1\right|=O\left(\frac{1}{\ln k}\right),\;k\to 0+,$$

which proves the expansion (2.7).

(iv) Moreover, $\omega (k)\in \sigma_{1}(k)$, as an eigenvalue of T(k) with multiplicity one, admits the representation

$$\omega (k)=\left(T(k)\varphi_{k},\varphi_{k}\right)=(T_{0}\varphi_{k},\varphi_{k})+\frac{1}{\ln k}(T_{1}\varphi_{k},\varphi_{k})+O\left(\frac{1}{\ln^{2}k}\right),\;k\to 0+.$$

Applying (2.7) and the fact that $T_{1}$ is bounded, we get

$$\omega (k)={\left|(\varphi ,\varphi_{k})\right|}^{2}+\frac{1}{\ln k}(T_{1}\varphi ,\varphi )+O\left(\frac{1}{\ln^{2}k}\right),\;k\to 0+.$$

Using (2.10) and (2.13) we get the asymptotics of ω(⋅) given in (2.8). □

### 2.3 Properties of the Q(⋅)-function

In this subsection we analyze the operator-valued function Q(⋅) defined in (2.5). Our aim is to describe certain basic properties of Q(⋅) and to derive its asymptotic expansion in the neighborhood of zero. The following lemma provides the first auxiliary tool.

Lemma 2.7 *Let μ be a compactly supported positive finite Radon measure on* $R^{2}$ *belonging to the generalized Kato class and let* C∈R *be a constant. Then the integral operator acting as*

$$Rf:=\int_{R^{2}}\left(-\ln\left(|\cdot -y|\right)+C\right)f(y)d\mu (y)$$

*is bounded in* $L_{\mu }^{2}$ *.*

Proof The operator *R* can be decomposed into the sum of two integral operators:

$$R_{1}f=\int_{R^{2}}\left(-\ln\left(|\cdot -y|\right)\right)f(y)d\mu (y),\;R_{2}f:=C\int_{R^{2}}f(y)d\mu (y).$$

According to the definition of the generalized Kato class (Definition 2.1) for any constant A>0 one can find ε>0 such that for every $x_{0}\in \mathrm{supp}\;\mu$ the estimate

(2.14) $$\int_{D_{\varepsilon }(x_{0})}\left|\ln\left(|x_{0}-y|\right)\right|d\mu (y)\leq A$$

holds. Hence for any $x_{0}\in \mathrm{supp}\;\mu$ we get

$$\int_{R^{2}}\left|\ln\left(|x_{0}-y|\right)\right|d\mu (y)=\int_{D_{\varepsilon }(x_{0})}\left|\ln\left(|x_{0}-y|\right)\right|d\mu (y)+\int_{R^{2}\setminus \bar{D_{\varepsilon }(x_{0})}}\left|\ln\left(|x_{0}-y|\right)\right|d\mu (y)\leq A+\max\left\{\left|\ln\left(|\varepsilon |\right)\right|,\left|\ln\left(|\mathrm{diam}\;\mathrm{supp}\;\mu |\right)\right|\right\}\mu_{T}.$$

Note that the above bound is independent of the choice of $x_{0}$ and therefore by the Schur criterion [43, Lemma 0.32] and the symmetry of the integral kernel the operator $R_{1}$ is bounded. Let $1_{\mu }$ stand for the identity function from $L_{\mu }^{2}$. Note that the integral operator $R_{2}$ is a rank-one operator $C1_{\mu }{\left(\cdot ,1_{\mu }\right)}_{L_{\mu }^{2}}$. Consequently, $R_{2}$ is also bounded. Now boundedness of *R* follows from decomposition $R=R_{1}+R_{2}$ and boundedness of $R_{1}$ and $R_{2}$ separately.  □

After these preliminaries we are ready to analyze the operator-valued function $R_{+}∋k\mapsto Q(-k^{2})$. First, let us note that for a given *k* the operator $Q(-k^{2})$ is bounded in $L_{\mu }^{2}$. The proof of this fact can be done via repeating the argument from [12, Corollary 2.2]. Now our aim is to expand Q(⋅) in a one-sided neighborhood of zero.

Proposition 2.8 *Let μ be a compactly supported positive Radon measure on* $R^{2}$ *from the generalized Kato class, and the operator-valued function* Q(⋅) *be defined as in* (2.5) *. Then* Q(⋅) *admits the expansion*

(2.15) $$Q\left(-k^{2}\right)=-\ln(k)P+R+O\left(k^{2}\ln(k)\right),\;k\to 0+,$$

*in the operator norm, where P is a rank-one operator given by*

(2.16) $$P:=\frac{1}{2\pi }1_{\mu }{\left(\cdot ,1_{\mu }\right)}_{L_{\mu }^{2}}$$

*and R is a bounded operator in* $L_{\mu }^{2}$ *defined by*

(2.17) $$Rf:=\frac{1}{2\pi }\int_{R^{2}}\left(-\ln|\cdot -y|-C_{E}+\ln2\right)f(y)d\mu (y);$$

$C_{E}$ *stands for the Euler–Mascheroni constant,* 1 *i.e.* $C_{E}=0.57721...$ *.*

Proof To prove the statement we employ the following expansion of the Macdonald function

(2.18) $$K_{0}(x)=-\ln(x/2)-C_{E}+s(x),\;x\to 0+,$$

where $s(x)=O(x^{2}\ln(x))$, see [1, Eq. (9.6.13)]. In view of (2.6) and the compactness of the support of *μ*, the operator $Q(-k^{2})$ can be expanded into the sum of the rank-one operator −ln⁡(k)P, the operator *R* and the remaining operator S(k) with the integral kernel s(k|x−y|). Since $Q(-k^{2})$, *P* and *R* are bounded the operator S(k) is bounded as well. Further, note that for sufficiently small k>0

$$\left|s\left(k|x-y|\right)\right|\leq A_{\mu }k^{2}\left|\ln(k)\right|,\;x,y\in \mathrm{supp}\;\mu ,$$

with some constant $A_{\mu }>0$, which depends on *μ*. Thus by Schur criterion the operator S(k) in $L_{\mu }^{2}$ with the integral kernel s(k|x−y|) satisfies

$$\left\|S(k)\right\|=O\left(k^{2}\ln k\right),\;k\to 0+,$$

which completes the proof.  □

Remark 2.9 Similar decomposition of the function Q(⋅) is employed in [14] for some other purposes in the case of Dirac measure supported by a non-compact curve.

In the next lemma we gather some useful properties of the operator-valued function Q(⋅).

Lemma 2.10 *Let the operator-valued function* Q(⋅) *be defined as in* (2.5) *. Then the following statements hold.*

(i) $Q(-k^{2})\geq 0$ *for all* k>0 *.*
(ii) $Q(-k_{1}^{2})\leq Q(-k_{2}^{2})$ *for* $k_{1}\geq k_{2}$ *.*
(iii) *For any* ε>0 *there exists sufficiently small* k>0 *such that the spectrum* $\sigma (Q(-k^{2}))$ *decomposes into two disjoint parts*
  $$\sigma_{0}\left(Q\left(-k^{2}\right)\right)\subset \left(0,\|R\|+\varepsilon \right)$$
  *with R as in* (2.17) *and*
  $$\sigma_{1}\left(Q\left(-k^{2}\right)\right)=\left\{\gamma (k)\right\},$$
  *where* γ(k) *is the eigenvalue of* $Q(-k^{2})$ *with multiplicity one.*
(iv) *The function* γ(⋅) *is continuous, strictly decaying, and* γ(k)→+∞ *as* k→0+ *.*

Proof The item (i) follows directly from the non-negativity of the Macdonald function and the representation of the integral kernel of $Q(-k^{2})$ given by (2.6), see also (2.27).

According to [12, Eq. (2.6)] one has that

(2.19) $$R_{\mu dx}\left(-{\overset{˜}{k}}^{2}\right)=R_{\mu dx}\left(-k^{2}\right)+\left({\overset{˜}{k}}^{2}-k^{2}\right)R\left(-{\overset{˜}{k}}^{2}\right)R_{\mu dx}\left(-k^{2}\right)$$

for any $k,\overset{˜}{k}\in R_{+}$. Hence, we arrive at the formula

(2.20) $$Q\left(-{\overset{˜}{k}}^{2}\right)-Q\left(-k^{2}\right)=\left({\overset{˜}{k}}^{2}-k^{2}\right){\left(R_{\mu dx}\left(-{\overset{˜}{k}}^{2}\right)\right)}^{⁎}R_{\mu dx}\left(-k^{2}\right);$$

where we combined (2.4) with (2.5), and used that $J_{\mu }R(-{\overset{˜}{k}}^{2})={\left(R_{\mu dx}(-{\overset{˜}{k}}^{2})\right)}^{⁎}$; cf. [7, Lemma 2.3]. Taking the adjoints in the formula (2.19) we get

$${\left(R_{\mu dx}\left(-{\overset{˜}{k}}^{2}\right)\right)}^{⁎}={\left(R_{\mu dx}\left(-k^{2}\right)\right)}^{⁎}+\left({\overset{˜}{k}}^{2}-k^{2}\right){\left(R_{\mu dx}\left(-k^{2}\right)\right)}^{⁎}R\left(-{\overset{˜}{k}}^{2}\right).$$

Inserting the above formula into (2.20) we obtain

(2.21) $$Q\left(-k^{2}\right)-Q\left(-{\overset{˜}{k}}^{2}\right)={\left(R_{\mu dx}\left(-k^{2}\right)\right)}^{⁎}\left[\left(k^{2}-{\overset{˜}{k}}^{2}\right)-{\left({\overset{˜}{k}}^{2}-k^{2}\right)}^{2}R\left(-{\overset{˜}{k}}^{2}\right)\right]R_{\mu dx}\left(-k^{2}\right).$$

Since $0\leq R(-{\overset{˜}{k}}^{2})\leq 1/({\overset{˜}{k}}^{2})$, we get if $k>\overset{˜}{k}$

$$\left(k^{2}-{\overset{˜}{k}}^{2}\right)-{\left({\overset{˜}{k}}^{2}-k^{2}\right)}^{2}R\left(-{\overset{˜}{k}}^{2}\right)\geq \left(k^{2}-{\overset{˜}{k}}^{2}\right)-\frac{{\left({\overset{˜}{k}}^{2}-k^{2}\right)}^{2}}{{\overset{˜}{k}}^{2}}=\left(k^{2}-{\overset{˜}{k}}^{2}\right)\frac{k^{2}}{{\overset{˜}{k}}^{2}}\geq 0.$$

The claim of (ii) follows directly from the last estimate and (2.21).

Note that according to Proposition 2.8 the function k↦T(k), k>0 defined by

(2.22) $$T(k):=-\frac{2\pi }{\mu_{T}\ln k}Q\left(-k^{2}\right)$$

determines a realization of the operator family considered in Theorem 2.6 with $H=L_{\mu }^{2}$, $\varphi =\frac{1_{\mu }}{\sqrt{\mu_{T}}}$ and $T_{1}=-\frac{2\pi }{\mu_{T}}R$ with *R* as in (2.17). Thus for sufficiently small k>0 the spectrum of the operator $Q(-k^{2})$ can be separated into two parts as claimed in (iii) and the function γ(⋅) is continuous. In view of (ii) the function γ(⋅) is non-increasing. Suppose that for some $k_{1}<k_{2}$ the condition $\gamma (k_{1})=\gamma (k_{2})$ holds, that implies γ(k)=c>0 for $k\in [k_{1},k_{2}]$. Hence, by Proposition 2.3 we have $\left[-k_{2}^{2},-k_{1}^{2}]\subset \sigma_{p}(H_{(1/c)\mu }\right)$ which leads to a contradiction since the point spectrum of any self-adjoint operator should be a countable set. This proves strict decay of γ(⋅). Furthermore, employing again (2.22) we conclude that $\gamma (k)=-\frac{\mu_{T}\ln k}{2\pi }\omega (k)$. Consequently, it follows from (2.8) that γ(k)→+∞ as k→0+.  □

### 2.4 Properties of the $R_{\mu dx}(\cdot )$-function

In this subsection we investigate some properties of the operator-valued function $R_{\mu dx}(\cdot )$ defined by (2.4). The unitary Fourier transform $F:L^{2}\to L^{2}$ is defined as the extension by continuity of the integral transform

$$(Ff)(p):=\frac{1}{2\pi }\int_{R^{2}}e^{-ipx}f(x)dx,\;f\in L^{2}\cap L^{1}.$$

It is well-known that F can be further extended by continuity up to the space $S^{\prime }$, cf. [2, Chapter 1.1.7]. Without a danger of confusion we keep the same notation $F:S^{\prime }\to S^{\prime }$ for this extension. In the following we will use also the abbreviation $Ff=\overset{ˆ}{f}$, $f\in S^{\prime }$. Applying again the standard results concerning the Sobolev spaces, see [2, Chapter 1.2.6], we can write

(2.23) $$H^{k}=\left\{f\in S^{\prime }:\overset{ˆ}{f}(p){\left(p^{2}+1\right)}^{k/2}\in L^{2}\right\},$$

where the norm ${\left\|\cdot \right\|}_{k}$ in $H^{k}$ is defined by ${\left\|f\right\|}_{k}=\|\overset{ˆ}{f}(p){\left(p^{2}+1\right)}^{k/2}\|L^{2}$. We define the functional *φμ* for $\varphi \in L_{\mu }^{2}$ as

$$(\varphi \mu )(f):=\int_{R^{2}}(J_{\mu }f)(x)\bar{\varphi (x)}d\mu (x),\;f\in H^{1},$$

with $J_{\mu }$ as in Section 2.1. Let us show that $\varphi \mu \in H^{-1}$. Indeed for any $f\in H^{1}$ we get

$$\left|(\varphi \mu )(f)\right|\leq \int_{R^{2}}\left|(J_{\mu }f)(x)\right|\left|\varphi (x)\right|d\mu (x)\leq {\left\|J_{\mu }f\right\|}_{L_{\mu }^{2}}{\left\|\varphi \right\|}_{L_{\mu }^{2}}\leq C{\left\|f\right\|}_{1}{\left\|\varphi \right\|}_{L_{\mu }^{2}}$$

with some constant C>0, where we applied Hölder inequality in between and used that the embedding $J_{\mu }$ of $H^{1}$ into $L_{\mu }^{2}$ is continuous. We have shown that the functional *φμ* is continuous on $H^{1}$ and hence $\varphi \mu \in H^{-1}$. Further, we define

(2.24) $$\overset{ˆ}{\varphi }(p):=\left(F(\varphi \mu )\right)(p)=\frac{1}{2\pi }\int_{R^{2}}e^{-ipx}\varphi (x)d\mu (x),\;\varphi \in L_{\mu }^{2}.$$

The above equivalence stays the extension of the Fourier transform to $L_{\mu }^{2}$; cf. [27]. In the next lemma we explore basic properties of the above transform.

Lemma 2.11 *Let μ be a compactly supported positive finite Radon measure on* $R^{2}$ *from the generalized Kato class. Then for any* $\varphi \in L_{\mu }^{2}$ *its Fourier transform* $\overset{ˆ}{\varphi }$ *given by* (2.24) *is a bounded and Lipschitz continuous function.*

Proof Let $\varphi \in L_{\mu }^{2}$. Since the measure *μ* is finite the inclusion $L_{\mu }^{2}\subset L_{\mu }^{1}$ holds. The boundedness of $\overset{ˆ}{\varphi }$ follows from the estimate

$${\left\|\overset{ˆ}{\varphi }\right\|}_{L^{\infty }}\leq \frac{1}{2\pi }{\left\|\varphi \right\|}_{L_{\mu }^{1}}<\infty .$$

It remains to show that $\overset{ˆ}{\varphi }$ is Lipschitz continuous. Let us choose arbitrary $p_{1},p_{2}\in R^{2}$. Applying (2.24) we obtain

(2.25) $$\left|\overset{ˆ}{\varphi }(p_{1})-\overset{ˆ}{\varphi }(p_{2})\right|\leq \frac{1}{2\pi }\int_{R^{2}}\left|e^{-ip_{1}x}-e^{-ip_{2}x}\right|\cdot \left|\varphi (x)\right|d\mu (x).$$

Using the fact that the function $R∋t\mapsto e^{it}$ is Lipschitz continuous we estimate

(2.26) $$\left|e^{-ip_{1}x}-e^{-ip_{2}x}\right|=\left|1-e^{-i(p_{2}-p_{1})x}\right|\leq L|x||p_{2}-p_{1}|$$

with some constant L>0. Plugging (2.26) into (2.25) and using that *μ* is compactly supported we get

$$\left|\overset{ˆ}{\varphi }(p_{1})-\overset{ˆ}{\varphi }(p_{2})\right|\leq L^{\prime }|p_{1}-p_{2}|$$

with some constant $L^{\prime }>0$.  □

Remark 2.12 Using the representation (2.23) of the Sobolev spaces we can extend operator $R(-k^{2})$ to a larger space. To derive this extension we apply

(2.27) $$R\left(-k^{2}\right)=F^{-1}\frac{1}{{\left|p\right|}^{2}+k^{2}}F:L^{2}\to L^{2},$$

cf. [2]. Operator $\frac{1}{{\left|p\right|}^{2}+k^{2}}F$ is bounded as the map acting from $H^{-1}$ to $L^{2}$ and, consequently, it can be extended by continuity to the whole space $H^{-1}$. This means that $R(-k^{2})$ admits the analogous extension. Note that $R_{\mu dx}(-k^{2})\varphi$ with $\varphi \in L_{\mu }^{2}$ can be identified with the extension of $R(-k^{2})$ defined above applied to $\varphi \mu \in H^{-1}$. To see this one can combine (2.24) and (2.27) into an iterated integral. After interchanging the order of integration and applying the integral representation [33, Eq. (19)] we get the claim due to (2.4).

In the next lemma we provide the Fourier representation of $R_{\mu dx}(-k^{2})$.

Lemma 2.13 *Let μ be a compactly supported positive finite Radon measure on* $R^{2}$ *from the generalized Kato class. The operator* $R_{\mu dx}(-k^{2}):L_{\mu }^{2}\to L^{2}$ *defined by* (2.4) *admits the representation*

(2.28) $$R_{\mu dx}\left(-k^{2}\right)\varphi =F^{-1}\frac{\overset{ˆ}{\varphi }(p)}{{\left|p\right|}^{2}+k^{2}},\;\varphi \in L_{\mu }^{2},$$

*where* $\overset{ˆ}{\varphi }$ *is given by* (2.24) *and* $F^{-1}$ *is the inverse Fourier transform on* $R^{2}$ *.*

Proof Combining the statements of Remark 2.12 and (2.24) we get the claim.  □

Having in mind later purposes we investigate in the next proposition the properties of $R_{\mu dx}(-k^{2})$ as k→0+.

Proposition 2.14 *Let μ be a compactly supported positive finite Radon measure on* $R^{2}$ *from the generalized Kato class. Let the operator-valued function* $R_{\mu dx}(-k^{2}):L_{\mu }^{2}\to L^{2}$ *be as in* (2.4) *. Then for any* $\varphi \in L_{\mu }^{2}$ *the following asymptotic expansion holds*

$$k^{2}{\left\|R_{\mu dx}\left(-k^{2}\right)\varphi \right\|}_{L^{2}}^{2}=\pi {\left|\overset{ˆ}{\varphi }(0)\right|}^{2}+O(\sqrt{k}),\;k\to 0+,$$

*where* $\overset{ˆ}{\varphi }$ *is the transform of φ defined by* (2.24) *.*

Proof Let $\varphi \in L_{\mu }^{2}$ and k>0. Using Lemma 2.13 and applying the fact that $F^{-1}$ is unitary in $L^{2}$ we obtain

(2.29) $$k^{2}{\left\|R_{\mu dx}\left(-k^{2}\right)\varphi \right\|}^{2}L^{2}=k^{2}\int_{R^{2}}\frac{{\left|\overset{ˆ}{\varphi }(p)\right|}^{2}}{{\left({\left|p\right|}^{2}+k^{2}\right)}^{2}}dp=\int_{R^{2}}\frac{{\left|\overset{ˆ}{\varphi }(kt)\right|}^{2}}{{\left({\left|t\right|}^{2}+1\right)}^{2}}dt.$$

We decompose the last integral of (2.29) onto regions

$$B_{k}=\left\{t\in R^{2}:|t|<\frac{1}{\sqrt{k}}\right\}\;\text{and}\;B_{k}^{c}=R^{2}\setminus \bar{B_{k}}.$$

Using boundedness of $\overset{ˆ}{\varphi }$ we obtain that

(2.30) $$\int_{B_{k}^{c}}\frac{{\left|\overset{ˆ}{\varphi }(kt)\right|}^{2}}{{\left({\left|t\right|}^{2}+1\right)}^{2}}dt\leq C\int_{B_{k}^{c}}\frac{1}{{\left({\left|t\right|}^{2}+1\right)}^{2}}dt=C^{\prime }\int_{\frac{1}{\sqrt{k}}}^{+\infty }\frac{r}{{\left(r^{2}+1\right)}^{2}}dr=O(k),\;k\to 0+.$$

Using boundedness and continuity of $\overset{ˆ}{\varphi }$, and applying mean-value theorem we arrive at

(2.31) $$\int_{B_{k}}\frac{{\left|\overset{ˆ}{\varphi }(kt)\right|}^{2}}{{\left({\left|t\right|}^{2}+1\right)}^{2}}dt={\left|\overset{ˆ}{\varphi }\left(\theta (k)\right)\right|}^{2}\int_{B_{k}}\frac{dt}{{\left({\left|t\right|}^{2}+1\right)}^{2}},$$

where $\theta (k)\in R^{2}$ and $|\theta (k)|\leq \sqrt{k}$. Applying the asymptotic behavior

$$\int_{B_{k}}\frac{dt}{{\left({\left|t\right|}^{2}+1\right)}^{2}}=\int_{R^{2}}\frac{dt}{{\left({\left|t\right|}^{2}+1\right)}^{2}}+O(k)=\pi +O(k),\;k\to 0+,$$

to the formula (2.31) we obtain

$$\int_{B_{k}}\frac{{\left|\overset{ˆ}{\varphi }(kt)\right|}^{2}}{{\left({\left|t\right|}^{2}+1\right)}^{2}}dt=\pi {\left|\overset{ˆ}{\varphi }\left(\theta (k)\right)\right|}^{2}+O(k),\;k\to 0+.$$

Lipschitz continuity and boundedness of $\overset{ˆ}{\varphi }$, and the inequality $\left|{\left|\overset{ˆ}{\varphi }(x)\right|}^{2}-{\left|\overset{ˆ}{\varphi }(x^{\prime })\right|}^{2}|\leq 2{\left\|\overset{ˆ}{\varphi }\right\|}_{L_{\mu }^{\infty }}|\overset{ˆ}{\varphi }(x)-\overset{ˆ}{\varphi }(x^{\prime })\right|$ combined with the above displayed formula, (2.29), (2.30) and $|\theta (k)|\leq \sqrt{k}$ imply that

$$k^{2}{\left\|R_{\mu dx}\left(-k^{2}\right)\varphi \right\|}_{L^{2}}^{2}=\pi {\left|\overset{ˆ}{\varphi }(0)\right|}^{2}+O(\sqrt{k}),\;k\to 0+,$$

and the claim is proven.  □

## 3 Weakly coupled bound state

In Section 3.1 we show that for sufficiently small coupling constant α>0 the discrete spectrum of the self-adjoint operator $H_{\alpha \mu }$ consists of exactly one negative eigenvalue of multiplicity one and we compute the asymptotics of this eigenvalue as α→0+. Moreover, in Section 3.2 we compute the asymptotics of the corresponding eigenfunction in the same limit.

### 3.1 Asymptotics of weakly coupled bound state

In this subsection we compute the asymptotics of weakly coupled bound state. The technique we employ here is slightly different than the one applied in [41]. As a benefit it allows to include also regular potentials with stronger singularities.

Theorem 3.1 *Let μ be a compactly supported positive finite Radon measure on* $R^{2}$ *from the generalized Kato class. Let the self-adjoint operator* $H_{\alpha \mu }$ *be as in* Definition 2.2 *. Then for all sufficiently small* α>0 *the condition*

$$♯\sigma_{d}(H_{\alpha \mu })=1$$

*holds and the corresponding unique eigenvalue* λ(α)<0 *satisfies*

(3.1) λ(α)→0−forα→0+.

Proof We rely on the Birman–Schwinger principle from Proposition 2.3. In order to recover the eigenvalues of $H_{\alpha \mu }$ we will investigate the following condition $1\in \sigma_{p}(\alpha Q(-k^{2}))$. Let $\sigma_{i}(Q(-k^{2}))$, i=0,1 be as in Lemma 2.10(iii). The possibility $1/\alpha \in \sigma_{0}(Q(-k^{2}))$ for k>0 small enough is excluded due to Lemma 2.10(iii). On the other hand, $1/\alpha \in \sigma_{1}(Q(-k^{2}))$ is equivalent to the equation

γ(k)=1/α,

which in view of Lemma 2.10(iv) has exactly one solution k(α) for α>0 small enough and moreover k(⋅) satisfies

k(α)→0+,α→0+.

Consequently, $\lambda (\alpha )=-k{\left(\alpha \right)}^{2}$ gives the unique negative simple eigenvalue of $H_{\alpha \mu }$ and the limiting property (3.1) holds.  □

Our next aim is to derive asymptotics of λ(α) for α→0+.

Theorem 3.2 *Let μ be a compactly supported positive finite Radon measure on* $R^{2}$ *from the generalized Kato class, and let* $H_{\alpha \mu }$ *be the self-adjoint operator as in* Definition 2.2 *. Then the eigenvalue corresponding to the weakly coupled bound state of* $H_{\alpha \mu }$ *admits the following asymptotics*

(3.2) $$\lambda (\alpha )=-\left(C_{\mu }+o(1)\right)\exp\left(-\frac{4\pi }{\alpha \mu_{T}}\right),\;\alpha \to 0+,$$

*where*

(3.3) $$C_{\mu }=\exp\left(\frac{4\pi }{\mu_{T}^{2}}{\left(R1_{\mu },1_{\mu }\right)}_{L_{\mu }^{2}}\right)=\exp\left(-2C_{E}+2\ln2-\frac{2}{\mu_{T}^{2}}\left(\int_{R^{2}}\int_{R^{2}}\ln|x-y|d\mu (x)d\mu (y)\right)\right).$$

Remark 3.3 Note that the error term appearing in (3.2) depends on *μ*. Therefore, in the following (especially in Section 4) we use the notation $o_{\mu }(1)$.

Proof of Theorem 3.2 Let us consider the operator-valued function

(3.4) $$T(k):=-\frac{2\pi }{\mu_{T}\ln k}Q\left(-k^{2}\right),\;k>0,$$

where Q(⋅) is defined by (2.5). Comparing the expansion from Proposition 2.8 and the definition (3.4) one can see that the operator-valued function T(⋅) reflects the structure assumed in Theorem 2.6; precisely $H=L_{\mu }^{2}$ and

$$T_{0}=\varphi_{\mu }{\left(\cdot ,\varphi_{\mu }\right)}_{L_{\mu }^{2}},\;T_{1}=-\frac{2\pi }{\mu_{T}}R,$$

where $\varphi \equiv \varphi_{\mu }:=\frac{1_{\mu }}{\sqrt{\mu_{T}}}$. Therefore, for sufficiently small k>0 the spectrum of T(k) can be separated into two disjoint parts: $\sigma_{0}(k)$ located in the neighborhood of 0 and $\sigma_{1}(k)$ consisting of exactly one simple eigenvalue ω(k) located in the neighborhood of 1 and admitting the asymptotic expansion

$$\omega (k)=1+\frac{1}{\ln k}{\left(T_{1}\varphi_{\mu },\varphi_{\mu }\right)}_{L_{\mu }^{2}}+O\left(\frac{1}{\ln^{2}k}\right),\;k\to 0+.$$

Applying the definition of $T_{1}$ to the last expansion, we arrive at

(3.5) $$\omega (k)=1-\frac{2\pi }{\mu_{T}^{2}\ln k}{\left(R1_{\mu },1_{\mu }\right)}_{L_{\mu }^{2}}+O\left(\frac{1}{\ln^{2}k}\right),\;k\to 0+.$$

Suppose that α>0 is sufficiently small, so that $♯\sigma_{d}(H_{\alpha \mu })=1$, cf. Theorem 3.1. Let $\lambda (\alpha )=-k^{2}(\alpha )$ standardly denote the corresponding unique eigenvalue of $H_{\alpha \mu }$ which in view of Theorem 3.1 converges as λ(α)→0− for α→0+. Combining the Birman–Schwinger principle together with the definition of T(⋅) we obtain the following condition

$$-\frac{1}{2\pi }\alpha \mu_{T}\omega \left(k(\alpha )\right)\ln k(\alpha )=1$$

for the value k(α). Applying to the above equation the asymptotic expansion of ω(⋅) given by (3.5) we get

$$-\frac{\alpha \mu_{T}\ln k(\alpha )}{2\pi }+\frac{\alpha }{\mu_{T}}{\left(R1_{\mu },1_{\mu }\right)}_{L_{\mu }^{2}}+O\left(\frac{\alpha }{\ln k(\alpha )}\right)=1,\;\alpha \to 0+.$$

The latter is equivalent to

(3.6) $$\ln k(\alpha )=-\frac{2\pi }{\alpha \mu_{T}}+\frac{2\pi }{\mu_{T}^{2}}{\left(R1_{\mu },1_{\mu }\right)}_{L_{\mu }^{2}}+o(1),\;\alpha \to 0+,$$

which yields

$$\lambda (\alpha )=-k{\left(\alpha \right)}^{2}=-\left(C_{\mu }+o(1)\right)e^{-\frac{4\pi }{\alpha \mu_{T}}},\;\alpha \to 0+,$$

with $C_{\mu }$ as in (3.3).  □

As a special case one gets the following statement.

Corollary 3.4 *Let* $\Sigma \subset R^{2}$ *be a finite piecewise* $C^{1}$ *curve being an open arc or a loop. Let L stand for the length of Σ and suppose that Σ is defined in the natural parametrization via the mapping* $\sigma :[0,L]\to R^{2}$ *. Let* $\mu_{\Sigma }$ *be the Dirac measure supported on Σ and let* $H_{\alpha \mu_{\Sigma }}$ *be the self-adjoint operator as in* Definition 2.2 *. Then the eigenvalue corresponding to the weakly coupled bound state of* $H_{\alpha \mu_{\Sigma }}$ *admits the following asymptotics*

$$\lambda (\alpha )=-\left(C_{\mu_{\Sigma }}+o(1)\right)\exp\left(-\frac{4\pi }{\alpha L}\right),\;\alpha \to 0+,$$

*with*

$$C_{\mu_{\Sigma }}=\exp\left(-2C_{E}+2\ln2-\frac{2}{L^{2}}\left(\int_{0}^{L}\int_{0}^{L}\ln\left|\sigma (s)-\sigma \left(s^{\prime }\right)\right|dsds^{\prime }\right)\right).$$

Example 3.1 We will test the above theorem on a special model. Namely, let *μ* be defined via the Dirac measure supported on the circle $C_{r}$ of radius *r*; precisely

(3.7) $$\mu (\Omega )=l(\Omega \cap C_{r}),$$

where l(⋅) is the one-dimensional measure defined by the length of the arc. This example was already studied in [24], where the authors compute negative spectrum of $H_{\alpha \mu }$ (with *μ* as above) using separation of variables.

In order to recover the asymptotic behavior of the eigenvalue of $H_{\alpha \mu }$ with *α* small and *μ* defined by (3.7), we will compute the constant $C_{\mu }$ given in (3.3). According to [32, Lemma 3.2] one has

(3.8) $${\left(Q\left(-k^{2}\right)1_{\mu },1_{\mu }\right)}_{L_{\mu }^{2}}=2\pi r^{2}\int_{0}^{\infty }\frac{{\left|J_{0}(y)\right|}^{2}y}{{\left(kr\right)}^{2}+y^{2}}dy,$$

where $J_{0}(\cdot )$ is the Bessel function of order 0. Applying [26, Eq. (6.535)] in the above formula we arrive at

(3.9) $${\left(Q\left(-k^{2}\right)1_{\mu },1_{\mu }\right)}_{L_{\mu }^{2}}=2\pi r^{2}I_{0}(kr)K_{0}(kr).$$

Using the asymptotic expansions [1, 9.6.12, 9.6.13]

$$I_{0}(x)=1+O\left(x^{2}\right),\;x\to 0+,$$

$$K_{0}(x)=-\left(\ln(x/2)+C_{E}\right)+O\left(x^{2}\ln x\right),\;x\to 0+,$$

of $I_{0}(\cdot )$ and $K_{0}(\cdot )$ in the neighborhood of zero, we obtain

(3.10) $$I_{0}(kr)K_{0}(kr)=-\ln\frac{kr}{2}-C_{E}+O(k),\;k\to 0+.$$

Combining Eqs. (3.9) and (3.10) we get

(3.11) $${\left(Q\left(-k^{2}\right)1_{\mu },1_{\mu }\right)}_{L_{\mu }^{2}}=2\pi r^{2}\left(-\ln\frac{kr}{2}-C_{E}+O(k)\right),\;k\to 0+.$$

The decomposition stated in Proposition 2.8 yields

(3.12) $${\left(R1_{\mu },1_{\mu }\right)}_{L_{\mu }^{2}}={\left(Q\left(-k^{2}\right)1_{\mu },1_{\mu }\right)}_{L_{\mu }^{2}}+2\pi r^{2}\ln k+O\left(k^{2}\ln k\right),\;k\to 0+.$$

In fact, the left hand side of (3.12) does not depend on *k*. Consequently, inserting (3.11) into (3.12) and taking the limit k→0+ we get

$${\left(R1_{\mu },1_{\mu }\right)}_{L_{\mu }^{2}}=2\pi r^{2}\left(-\ln\frac{r}{2}-C_{E}\right).$$

In view of (3.3) this implies that $C_{\mu }=\frac{4}{r^{2}}\exp(-2C_{E})$ and finally

$$\lambda (\alpha )=-\frac{4}{r^{2}}e^{-2C_{E}}e^{-\frac{2}{\alpha r}}\left(1+o(1)\right),\;\alpha \to 0+,$$

which is consistent with a result of [24, Section 2.1] up to the factor $\exp(-2C_{E})$. Note that this factor can be also obtained by the method applied in [24].

Remark 3.5 Following the approach of [12] one can introduce a sign changing weight in $\gamma \in L^{\infty }(R^{2})$ and consider more general operators defined via quadratic forms

$$q_{\alpha \gamma \mu }[f]:={\left\|\nabla f\right\|}_{L^{2}}^{2}-\alpha \int_{R^{2}}\gamma (x){\left|f(x)\right|}^{2}d\mu (x),\;\mathrm{dom}\;q_{\alpha \gamma \mu }=H^{1}.$$

Then we may expect that the existence of a negative eigenvalue of $H_{\alpha \gamma \mu }$ depends on sign of $I:=\int_{R^{2}}\gamma (x)d\mu (x)$. If I>0 then $H_{\alpha \gamma \mu }$ admits a negative eigenvalue with analogous asymptotics as (3.2) with *I* playing a role of $\mu_{T}$ in the exponent. The asymptotics could be different if I=0. However, the analysis of sign changing case needs either an extension of Theorem 2.6 for non-self-adjoint operators or modifying the formulation of the Birman–Schwinger principle stated in Proposition 2.3. We postpone this for further studies.

### 3.2 Asymptotics of the eigenfunction corresponding to the weakly coupled bound state

As we have shown in the previous section the operator $H_{\alpha \mu }$ has exactly one negative eigenvalue for sufficiently small α>0. The aim of this section is to recover the asymptotic behavior of the corresponding eigenfunction in the limit α→0+.

Theorem 3.6 *Let μ be a compactly supported positive finite Radon measure on* $R^{2}$ *from the generalized Kato class. Let* λ(α)<0 *be the unique eigenvalue of* $H_{\alpha \mu }$ *in the limit* α→0+ *and set* $k_{\alpha }:=\sqrt{-\lambda (\alpha )}$ *. Then the corresponding eigenfunction has the form*

$$f_{\alpha }(\cdot )=\frac{k_{\alpha }}{2\pi }\int_{R^{2}}K_{0}\left(k_{\alpha }|\cdot -y|\right)d\mu (y)+O\left(\frac{1}{\ln k_{\alpha }}\right),\;\alpha \to 0+,$$

*where the error term is understood in the sense of* $L^{2}$ *-norm; moreover the* $L^{2}$ *-norm of* $f_{\alpha }$ *has non-zero finite limit as* α→0+ *.*

Proof In the proof of this theorem we rely on Proposition 2.3. For non-trivial $\phi_{\alpha }\in \ker(I-\alpha Q(-k_{\alpha }^{2}))$ the function

(3.13) $$g_{\alpha }(\cdot ):=R_{\mu dx}\left(-k_{\alpha }^{2}\right)\phi_{\alpha }=\frac{1}{2\pi }\int_{R^{2}}K_{0}\left(k_{\alpha }|\cdot -y|\right)\phi_{\alpha }(y)d\mu (y),$$

reproduces the eigenfunction of $H_{\alpha \mu }$. Similarly as in the proof of Theorem 3.2 we conclude that $\phi_{\alpha }$ is an eigenfunction of the operator

$$T(k_{\alpha })=-\frac{2\pi }{\mu_{T}\ln(k_{\alpha })}Q\left(-k_{\alpha }^{2}\right)$$

corresponding to the eigenvalue $-\frac{2\pi }{\mu_{T}\ln(k_{\alpha })\alpha }$. Recall that the family $R_{+}∋k\mapsto T(k)$ is a realization of the operator family considered in Theorem 2.6 with $H=L_{\mu }^{2}$, $\varphi :=\frac{1_{\mu }}{\sqrt{\mu_{T}}}$, $T_{0}:=\varphi (\cdot ,\varphi )$, $T_{1}:=-\frac{2\pi }{\mu_{T}}R$ and *R* as in (2.17). Hence, by Theorem 2.6(iii) we obtain that $\phi_{\alpha }$ can be chosen in the form

(3.14) $$\phi_{\alpha }=1_{\mu }+\varsigma_{\alpha },\;\text{where }{\left\|\varsigma_{\alpha }\right\|}_{L_{\mu }^{2}}=O\left(\frac{1}{\ln k_{\alpha }}\right)\text{ as }\alpha \to 0+.$$

By Proposition 2.14 we obtain

(3.15) $$k_{\alpha }^{2}{\left\|R_{\mu dx}\left(-k_{\alpha }^{2}\right)1_{\mu }\right\|}^{2}L^{2}=\frac{\mu_{T}^{2}}{4\pi }+O(\sqrt{k_{\alpha }}),\;\alpha \to 0+,$$

where we used that ${\overset{ˆ}{1}}_{\mu }(0)=\frac{1}{2\pi }\mu_{T}$. Hölder inequality yields

(3.16) $$\left|{\overset{ˆ}{\varsigma }}_{\alpha }(0)\right|\leq \frac{1}{2\pi }{\left\|\varsigma_{\alpha }\right\|}_{L_{\mu }^{1}}\leq \frac{1}{2\pi }{\left\|\varsigma_{\alpha }\right\|}_{L_{\mu }^{2}}\sqrt{\mu_{T}}.$$

Hence, using Proposition 2.14, (3.14) and (3.16) we get

(3.17) $$k_{\alpha }^{2}{\left\|R_{\mu dx}\left(-k_{\alpha }^{2}\right)\varsigma_{\alpha }\right\|}^{2}L^{2}=O\left(\frac{1}{\ln^{2}k_{\alpha }}\right),\;\alpha \to 0+.$$

According to (3.13), (3.14), (3.15) and (3.17)

$$f_{\alpha }:=k_{\alpha }R_{\mu dx}\phi_{\alpha }$$

is an eigenfunction of $H_{\alpha \mu }$ and satisfies

$${\left\|f_{\alpha }\right\|}_{L^{2}}\to \frac{\mu_{T}}{2\sqrt{\pi }},\;\text{as }\alpha \to 0+,$$

moreover

$$f_{\alpha }(\cdot )=\frac{k_{\alpha }}{2\pi }\int_{R^{2}}K_{0}\left(k_{\alpha }|\cdot -y|\right)d\mu (y)+O\left(\frac{1}{\ln k_{\alpha }}\right),\;\alpha \to 0+,$$

holds, and the claim is proven.  □

As a special case one gets the following statement.

Corollary 3.7 *Let* $\Sigma \subset R^{2}$ *be a finite piecewise* $C^{1}$ *curve being an open arc or a loop. Let L stand for the length of Σ and suppose that Σ is defined in the natural parametrization via the mapping* $\sigma :[0,L]\to R^{2}$ *. Let* $\mu_{\Sigma }$ *be the Dirac measure supported on Σ and let* $H_{\alpha \mu_{\Sigma }}$ *be the self-adjoint operator as in* Definition 2.2 *. Let* λ(α)<0 *be the unique eigenvalue of* $H_{\alpha \mu }$ *in the limit* α→0+ *and set* $k_{\alpha }:=\sqrt{-\lambda (\alpha )}$ *. Then the corresponding eigenfunction has the form*

$$f_{\alpha }(\cdot )=\frac{k_{\alpha }}{2\pi }\int_{\Sigma }K_{0}\left(k_{\alpha }|\cdot -y|\right)d\sigma (y)+O\left(\frac{1}{\ln k_{\alpha }}\right),\;\alpha \to 0+,$$

*where σ is the natural Lebesgue measure on Σ, the error term is understood in the sense of* $L^{2}$ *-norm; moreover the* $L^{2}$ *-norm of* $f_{\alpha }$ *has non-zero finite limit as* α→0+ *.*

## 4 Remarks on non-compactly supported finite measures

In this section we show that the asymptotics of the type (3.2) holds also for some non-compactly supported finite Kato class measures. Precisely, we derive a lower and an upper bound for the lowest eigenvalue in the weak coupling constant regime. Furthermore, we show that the results obtained here allow to recover an information on the weak coupling constant asymptotics in cases for which previously known results are not applicable.

Theorem 4.1 *Let μ be a finite positive Radon measure on* $R^{2}$ *from the generalized Kato class (not necessarily compactly supported). Let the self-adjoint operator* $H_{\alpha \mu }$ *be as in* Definition 2.2 *. Then for each* α>0 *the operator* $H_{\alpha \mu }$ *has at least one negative eigenvalue. Moreover, for any sufficiently small* ε>0 *there exist* $\alpha_{\varepsilon }>0$ *and* C(ε)>0 *such that for* $\alpha \in (0,\alpha_{\varepsilon })$ *the lowest eigenvalue* λ(α)<0 *of* $H_{\alpha \mu }$ *satisfies the estimate*

$$\lambda (\alpha )\leq -C(\varepsilon )\exp\left(-\frac{4\pi }{\alpha (\mu_{T}-\varepsilon )}\right),\;\alpha \in (0,\alpha_{\varepsilon }).$$

Proof In view of Proposition 2.4 one has $\sigma_{\text{ess}}(H_{\alpha \mu })=[0,+\infty )$. Let us fix r>0 and take the restriction $\mu_{r}$ of the measure *μ* onto the disc $D_{r}(0):=\{(x,y)\in R^{2}:x^{2}+y^{2}<r^{2}\}$. Consequently, $\inf\sigma (H_{\alpha \mu_{r}})\geq \inf\sigma (H_{\alpha \mu })$. Thus the operator $H_{\alpha \mu }$ has at least one negative eigenvalue for all α>0, because the operator $H_{\alpha \mu_{r}}$ has and because $\sigma_{\text{ess}}(H_{\alpha \mu })=[0,+\infty )$.

Observe that for any sufficiently small ε>0 there exists a sufficiently large r(ε)>0 such that $\mu_{T}-\mu_{r(\varepsilon )}(R^{2})=\varepsilon$. Set $C(\varepsilon ):=C_{\mu_{r(\varepsilon )}}+\sup_{\alpha \in (0,\alpha_{\varepsilon })}r_{\mu_{r(\varepsilon )}}(\alpha )$, where $C_{\mu_{r(\varepsilon )}}$ and $r_{\mu_{r(\varepsilon )}}(\alpha ):=o_{\mu_{r(\varepsilon )}}(1)$ are determined by (3.2) and (3.3). Finally, Theorem 3.2 implies the statement.  □

Remark 4.2 The following two aspects should be mentioned.

(a) It is not claimed in Theorem 4.1 that for any non-compactly supported finite measure from the generalized Kato class the operator $H_{\alpha \mu }$ has exactly one bound state for all sufficiently small α>0.
(b) Assume that *μ* satisfies
  (4.1) $$\left|\int_{R^{2}}\int_{R^{2}}\ln\left(|x-y|\right)d\mu (x)d\mu (y)\right|<\infty .$$
  Then C(ε)>0 in the previous theorem can be chosen for sufficiently small α>0 arbitrarily close to $C_{\mu }$ defined by (3.3).

Hypothesis 4.1 *Assume that μ is a finite Kato class measure on* $R^{2}$ *, which is not necessarily compactly supported. Suppose that* ${\left\{\mu_{n}\right\}}_{n=1}^{\infty }$ *are compactly supported finite measures from the generalized Kato class such that* $\mu_{n}(R^{2})=\mu_{T}$ *for all* n∈N *and that* $\mu =\sum_{n=1}^{\infty }a_{n}\mu_{n}$ *, where* $a_{n}>0$ *(* n∈N *) and* $\sum_{n=1}^{\infty }a_{n}=1$ *. Assume also that*

(4.2) $$C^{⁎}:=\sum_{n=1}^{\infty }a_{n}C_{\mu_{n}}<\infty \;\text{and}\;\sum_{n=1}^{\infty }a_{n}r_{\mu_{n}}(\alpha )=o(1),\;\alpha \to 0+,$$

*where* $r_{\mu_{n}}(\alpha ):=o_{\mu_{n}}(1)$ *and* $C_{\mu_{n}}$ *are defined by* (3.2) *and* (3.3) *, respectively.*

Remark 4.3 If the family ${\left\{\mu_{n}\right\}}_{n=1}^{\infty }$ in Hypothesis 4.1 consists of infinitely many (possibly rotated and translated) copies of compactly supported measures from a finite set, then the conditions of Hypothesis 4.1 are naturally satisfied.

Theorem 4.4 *Let μ be a finite positive Radon measure on* $R^{2}$ *from the generalized Kato class satisfying* Hypothesis 4.1 *, where* $C^{⁎}$ *is as in* (4.2) *. Let the self-adjoint operator* $H_{\alpha \mu }$ *be as in* Definition 2.2 *. Let* λ(α)<0 *be the lowest eigenvalue of* $H_{\alpha \mu }$ *. Then*

$$\lambda (\alpha )\geq -\left(C^{⁎}+o(1)\right)\exp\left(-\frac{4\pi }{\alpha \mu_{T}}\right),\;\alpha \to 0+.$$

Proof Let us pick a function $f\in H^{1}$. Plugging this function into the form $t_{\alpha \mu }$ in (2.3) and estimating with the aid of Theorem 3.2, we get

$$t_{\alpha \mu }[f,f]={\left\|\nabla f\right\|}_{L^{2}}^{2}-\alpha {\left\|J_{\mu }f\right\|}_{L_{\mu }^{2}}^{2}=\sum_{n=1}^{\infty }a_{n}\left({\left\|\nabla f\right\|}_{L^{2}}^{2}-\alpha {\left\|J_{\mu_{n}}f\right\|}_{L_{\mu_{n}}^{2}}^{2}\right)\geq -\sum_{n=1}^{\infty }a_{n}\left(C_{\mu_{n}}+r_{\mu_{n}}(\alpha )\right)e^{-\frac{4\pi }{\alpha \mu_{T}}}{\left\|f\right\|}_{L^{2}}^{2}=-\left(C^{⁎}+o(1)\right)e^{-\frac{4\pi }{\alpha \mu_{T}}}{\left\|f\right\|}_{L^{2}}^{2},$$

and the claim follows by variational principles.  □

Remark 4.5 Note that with the aid of Theorem 4.1, Theorem 4.4 one can obtain information on the asymptotics of the lowest eigenvalue also in those cases, where the results of [8], [41] are not applicable. For instance, we do not need the second condition in (1.1) for regular potentials. However, it should be said that for our class of non-compactly supported measures we are able only to recover the first term in the asymptotic expansion, covering [41] and not [8]. Also, in general, verifying the conditions of Hypothesis 4.1 is not very easy, except an important case indicated in Remark 4.3.

Example 4.1 Let us split the plane into squares ${\left\{\Pi_{n}\right\}}_{n=1}^{\infty }$ with the unit side i.e. $R^{2}=\bar{{⋃}_{n=1}^{\infty }\Pi_{n}}$ and $\Pi_{n}\cap \Pi_{m}=\emptyset$ for n≠m. We denote by $\chi_{\Pi_{n}}(\cdot )$ the characteristic function of $\Pi_{n}$. Then the potential

$$V(x):=\sum_{n=1}^{\infty }\frac{1}{n\ln^{2}(n+1)}\chi_{\Pi_{n}}(x),$$

belongs to the generalized Kato class and the corresponding measure $\mu_{V}$ is finite since $\mu_{V}(R^{2})=\sum_{n=1}^{\infty }\frac{1}{n\ln^{2}(n+1)}<\infty$. The potential *V* does not satisfy the second condition in (1.1) and thus the results of [8], [41] are not applicable, but the measure $\mu_{V}$ satisfies Hypothesis 4.1 with $a_{n}=\frac{1}{n\ln^{2}(n+1)\mu_{V}(R^{2})}$ and $\mu_{n}$ being defined as

$$\mu_{n}(\Omega ):=\mu_{V}\left(R^{2}\right)\mathrm{Area}(\Omega \cap \Pi_{n}),$$

which is in fact for different n∈N the same measure (up to translations). By Proposition 2.4 one has that $\sigma_{\text{ess}}(H_{\alpha \mu_{V}})=[0,+\infty )$, and by Theorem 4.1 the operator $H_{\alpha \mu_{V}}$ has at least one negative eigenvalue for all α>0. Let λ(α)<0 be the lowest eigenvalue of $H_{\alpha \mu_{V}}$. Employing Theorem 4.1, Theorem 4.4 we arrive at

$$-\left(C^{⁎}+o(1)\right)\exp\left(-\frac{4\pi }{\alpha \mu_{T}}\right)\leq \lambda (\alpha )\leq -C(\varepsilon )\exp\left(-\frac{4\pi }{\alpha (\mu_{T}-\varepsilon )}\right),\;\alpha \in (0,\alpha_{\varepsilon }),$$

for arbitrarily small ε>0 with some constants $\alpha_{\varepsilon },C^{⁎},C(\varepsilon )>0$ as in the utilized theorems.

Example 4.2 Consider a half-line in $R^{2}$ defined by $\Sigma :=\{(s,0):s\in R_{+}\}$. For any n∈N define the unit segment $\Sigma_{n}:=[n-1,n)$, i.e. $\Sigma ={⋃}_{n=1}^{\infty }\Sigma_{n}$. Assume that the potential-measure takes the form

$$\mu :=\sum_{n=1}^{\infty }\frac{1}{1+n^{1+\varepsilon }}\delta_{\Sigma_{n}},\;\varepsilon >0,$$

where $\delta_{\Sigma_{n}}$ standardly denotes the Dirac *δ*-function supported on $\Sigma_{n}$. Therefore, $\mu (R^{2})=\sum_{n=1}^{\infty }\frac{1}{1+n^{1+\varepsilon }}<\infty$ and, consequently, $\sigma_{\text{ess}}(H_{\alpha \mu })=[0,\infty )$. Introduce

$$\mu_{n}:=\mu \left(R^{2}\right)\delta_{\Sigma_{n}}\;\text{and}\;a_{n}:=\frac{1}{\left(1+n^{1+\epsilon })\mu (R^{2}\right)}.$$

Let λ(α)<0 the lowest eigenvalue of $H_{\alpha \mu }$. Applying Theorem 4.1, Theorem 4.4 we obtain that

(4.3) $$-\left(C^{⁎}+o(1)\right)\exp\left(-\frac{4\pi }{\alpha \mu_{T}}\right)\leq \lambda (\alpha )\leq -C(\varepsilon )\exp\left(-\frac{4\pi }{\alpha (\mu_{T}-\varepsilon )}\right),\;\alpha \in (0,\alpha_{\varepsilon }),$$

for arbitrarily small ε>0 with some constants $\alpha_{\varepsilon },C^{⁎},C(\varepsilon )>0$ as in the utilized theorems. Consider now the measure

$$\overset{˘}{\mu }=\frac{1}{1+{\left|s+1\right|}^{1+\varepsilon }}\delta_{\Sigma }.$$

Using the inequality

$${\left\|J_{\mu }f\right\|}_{L_{\mu }^{2}}\geq {\left\|J_{\overset{˘}{\mu }}f\right\|}_{L_{\overset{˘}{\mu }}^{2}},\;f\in H^{1},$$

and employing the argument used in the proof of Theorem 4.4 we conclude that the expression $-(C^{⁎}+o(1))\exp(-\frac{4\pi }{\alpha \mu_{T}})$ yields a lower bound for the lowest eigenvalue of $H_{\alpha \overset{˘}{\mu }}$. Moreover, changing $\mu_{T}$ by ${\overset{˘}{\mu }}_{T}=\overset{˘}{\mu }(R^{2})$ in the upper bound in (4.3) we get a corresponding upper bound of the lowest eigenvalue for the measure $\overset{˘}{\mu }$.

## Footnotes

1 This constant can be computed as $C_{E}=\lim_{n\to \infty }(\sum_{k=1}^{n}\frac{1}{k}-\ln(n))$.
